# Quantitative anatomy of the primary ossification center in the fetal pubis bone

**DOI:** 10.1007/s00276-019-02229-4

**Published:** 2019-03-29

**Authors:** Mariusz Baumgart, Marcin Wiśniewski, Magdalena Grzonkowska, Mateusz Badura, Michał Szpinda, Katarzyna Pawlak-Osińska

**Affiliations:** 1 Department of Normal Anatomy, The Ludwik Rydygier Collegium Medicum in Bydgoszcz, The Nicolaus Copernicus University in Toruń, Łukasiewicza 1 Street, 85-821 Bydgoszcz, Poland; 2 Department of Otolaryngology and Oncology, The Ludwik Rydygier Collegium Medicum in Bydgoszcz, The Nicolaus Copernicus University in Toruń, Bydgoszcz, Poland

**Keywords:** Pubis, Bone development, Osteogenesis, Fetal development

## Abstract

**Purposes:**

Skeletodysplasiae and hereditary dysostoses constitute a group of over 350 disorders of the skeletal system. Knowledge about development of the pubic primary ossification center may be useful in both determining the fetal stage and maturity, and for detecting congenital disorders. The present study was performed to quantitatively examine the pubic primary ossification center with respect to its linear, planar, and volumetric parameters.

**Materials and methods:**

Using methods of computed tomography (CT), digital-image analysis and statistics, the size of the pubic primary ossification center in 33 spontaneously aborted human fetuses (18 males and 15 females) aged 22–30 weeks was studied.

**Results:**

With no sex and laterality differences, the best-fit growth dynamics for the pubic primary ossification center was modeled by the following functions: *y* = − 13.694 + 0.728 × age ± 0.356 for its sagittal diameter, *y* = − 3.350 + 0.218 × age ± 0.159 for its vertical diameter, *y* = − 61.415 + 2.828 × age ± 1.519 for its projection surface area, and *y* = − 65.801 + 3.173 × age ± 2.149 for its volume.

**Conclusions:**

The size of the pubic primary ossification center shows neither sex nor laterality differences. The growth dynamics of the vertical and sagittal diameters, projection surface area, and volume of the pubic ossification centers follow proportionately to fetal age. The obtained numerical findings of the pubic ossification center are considered age-specific reference data with clinical implications in the diagnostics of congenital defects.

## Introduction

Skeletodysplasiae and genetic dysostoses are a group of over 350 disorders of the skeletal system. Of note, inasmuch as the term skeletodysplasiae refers to generalized disorders of the skeletal system, the naming dysostoses encompasses abnormalities of one to several bones. Although individual skeletodysplasiae are sporadic, they occur in a significant number of neonates with congenital anomalies, many of which are lethal, and their incidence is estimated at approximately 1/50,000–1/20,000 births. The fetal skeletal system develops as early as in weeks 7–8 of gestation, which allows detecting a skeletal defect during routine ultrasonic examinations [18].

Primary ossification centers can ultrasonically be visualized starting from week 9 of gestation, reaching the detectability of lethal skeletodysplasiae at the range of 94–96% [17]. Primary ossification centers appear in the first trimester of pregnancy, between weeks 7 and 12, while secondary ossification centers refer to the second and third trimesters of pregnancy [17, 19].

Although the timing of ossification of each constituent of the hip bone has precisely been recognized, no measurements of the pubic ossification centers have been reported in the medical literature. In addition, detailed morphometric data on the development of ossification centers in human fetuses can be useful in the early detection of skeletodysplasiae associated with a delayed development of ossification centers and their mineralization [23].

This is the first report in the literature to display the morphometric analysis of the pubic primary ossification centers in human fetuses based on computed tomography (CT) imaging. This study is a continuation of our research on the development of the pelvic girdle and lower limb bones [2, 3].

The purposes of this study were:

- to perform morphometric analysis of the pubic ossification center in human fetuses (linear, superficial, and spatial parameters) to determine their normative value;
- to examine possible differences between sexes for all analyzed parameters;
- to compute development dynamics for the analyzed parameters, expressed by best-matched mathematical models.

## Materials and methods

The study material was 33 human fetuses (18 males and 15 females) aged 22–30 weeks of fetal life, derived from spontaneous miscarriages and preterm deliveries. The fetuses were acquired before the year 2000 and remain part of the specimen collection of Department of Normal Anatomy, The Ludwik Rydygier Collegium Medicum in Bydgoszcz, and The Nicolaus Copernicus University in Toruń, Poland. The experiment was sanctioned by the Bioethics Committee (KB 275/2011). The fetal ages (Table 1) were determined based on the crown–rump length (CRL).

**Table 1:** Age, number, and sex of the fetuses studied

Age (weeks)	Crown–rump length (mm)				Number of fetuses	Sex	
	Mean	SD	Min	Max		♂	♀
22	185.00	2.12	183.00	187.00	2	0	2
23	198.67	2.89	197.00	202.00	3	1	2
24	209.44	3.68	204.00	213.00	9	5	4
25	215.50	2.12	214.00	217.00	2	1	1
26	225.00	0.82	224.00	226.00	4	2	2
27	237.75	2.75	235.00	241.00	4	4	0
28	246.67	4.93	241.00	250.00	3	1	2
29	255.00	2.00	253.00	257.00	3	2	1
30	264.66	1.15	263.00	266.00	3	2	1
Total					33	18	15

**Fig. 1:**
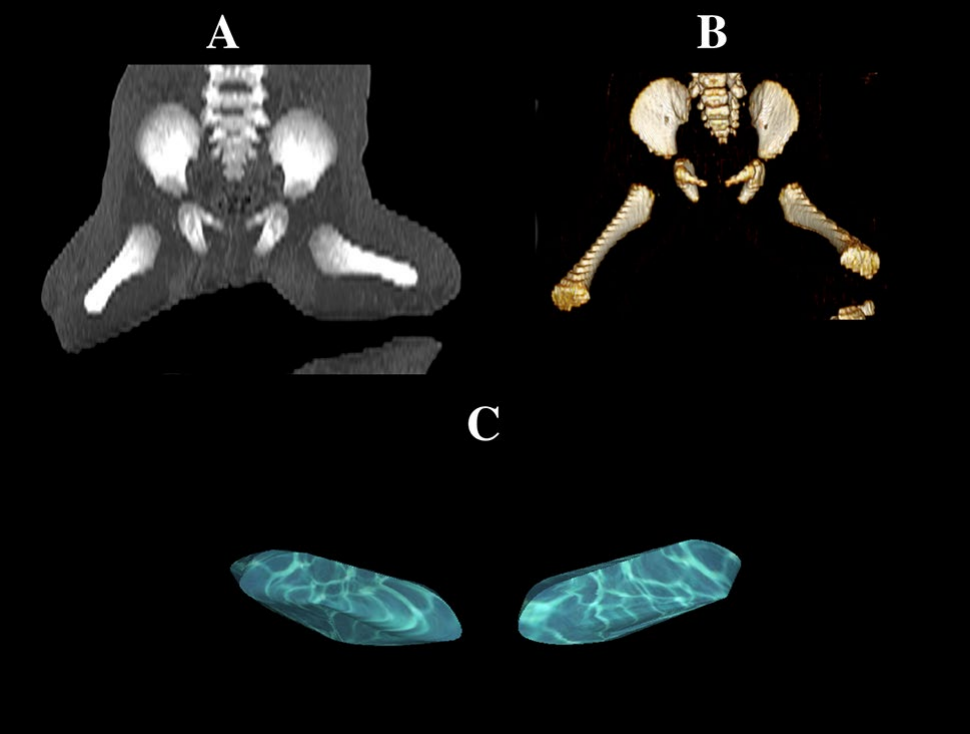
CT of the pelvis of the human fetus in the frontal projection (**a**), 3D reconstruction of the pelvis in the frontal projection (**b**), and primary ossification center of the pubis (**c**) using Osirix 3.9

With the use of a Siemens-Biograph 128 mCT camera (Siemens Healthcare GmbH, Erlangen, Germany) placed at Department of Positron Emission Tomography and Molecular Imaging (Oncology Center, Collegium Medicum of the Nicolaus Copernicus University, Bydgoszcz, Poland), all fetuses were scanned at a step of 0.4 mm, recorded in DICOM formats (Fig. 1), and successively subjected to morphometric analysis using the Osirix 3.9 software. Delineations of the pubic ossification center were evidently visible [6, 11], thus, enabling us to perform morphometric analysis in terms of its linear, planar, and spatial parameters.

Technical parameters of achieved CT images were as follows: gray scale ranged from − 275 to – 134 HU for a minimum, and from + 1165 to + 1558 HU for a maximum, window width alternated from 1.404 to 1.692, window level varied from + 463 to + 712, mAs = 60, kV = 80, pitch = 0.35, FoV = 180, rotation time = 0.5 s., slice thickness = 0.4 mm, image increment = 0.6 mm, and kernel = B45 f-medium.

**Fig. 2:**
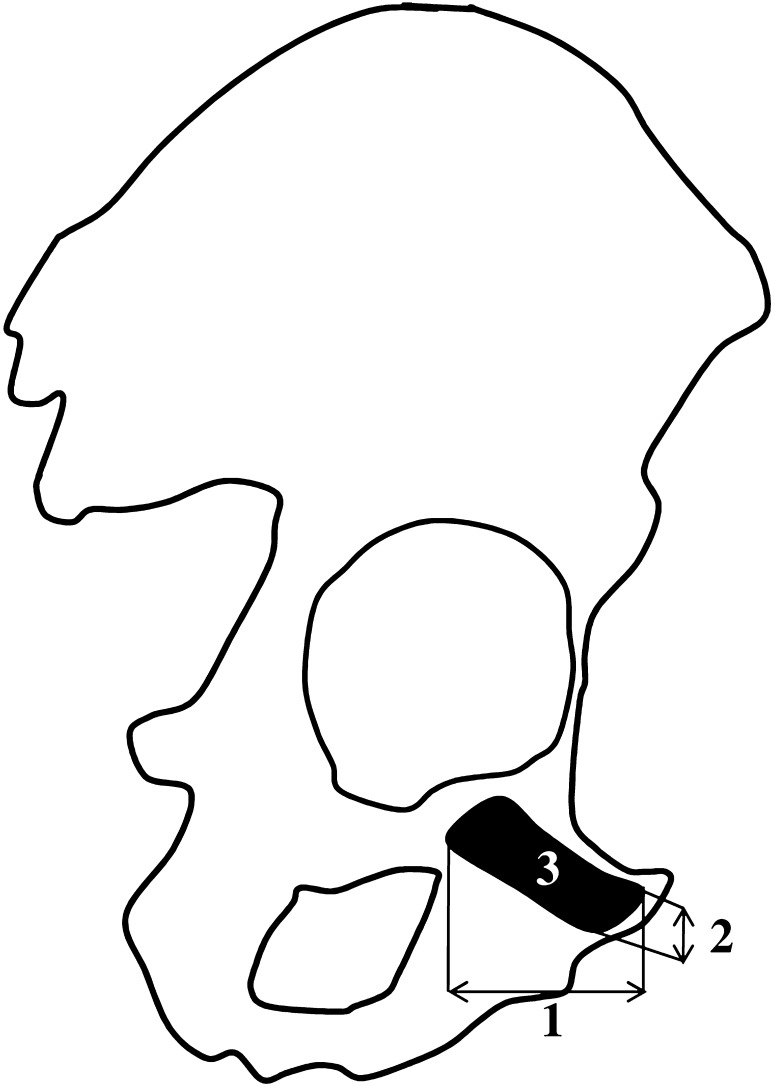
Measurement scheme of the pubic primary ossification center in the frontal plane. 1—Sagittal diameter, 2—vertical diameter, and 3—projection surface area

The following four measurements of every pubic ossification center were conducted in a specific order (Fig. 2) in 33 fetuses, including:

1. sagittal diameter, based on the determined distance between the anterior and posterior borderlines of the pubic ossification center in the sagittal plane (Fig. 2);
2. vertical diameter, based on the determined distance between the superior and inferior borderlines of the ossification center in the sagittal plane (Fig. 2);
3. projection surface area, based on the determined contour of the pubic ossification center in the sagittal plane (Fig. 2); and
4. volume, calculated using advanced diagnostic imaging tools for 3D reconstruction, taking into account position and the absorption of radiation by bone tissue (Fig. 1c).

The numerical results were statistically analyzed. Distribution of variables was checked using the Shapiro–Wilk (*W*) test, while homogeneity of variance was checked using Fisher’s test. The results were expressed as arithmetic means ± standard deviations (SD). To compare the means, Student’s *t* test for independent variables and ANOVA were used. Tukey’s test was used for post hoc analysis. If no similarity of variance occurred, the non-parametric Kruskal–Wallis test was used. The growth dynamics of the analyzed parameters was based on linear and non-linear regression analyses. The match between the estimated curves and measurement results was evaluated based on the coefficient of determination (*R*^2^).

## Results

The statistical analysis revealed neither significant sex nor bilateral differences, allowing us to compute only one growth curve for each analyzed parameter. Mean values and standard deviations for sagittal and vertical diameters, projection surface area, and volume of the pubic ossification centers at varying gestational ages for the right (Table 2) and left (Table 3) sides have been presented.

**Table 2:** Sagittal and vertical diameters, projection surface area, and volume of the ossification centers of the right pubis

Gestational age (weeks)	Number of fetuses	Ossification centers of the right pubis							
		Sagittal diameter (mm)		Vertical diameter (mm)		Projection surface area (mm^2^)		Volume (mm^3^)	
		Mean	SD	Mean	SD	Mean	SD	Mean	SD
22	2	2.31	0.03	1.26	0.01	3.17	0.10	7.09	0.48
23	3	2.82	0.42	1.52	0.19	4.14	0.47	7.87	0.22
24	9	3.69	0.43	1.91	0.20	5.66	1.06	9.62	0.58
25	2	4.40	0.04	2.15	0.01	7.59	0.10	11.07	0.99
26	4	4.97	0.27	2.37	0.01	9.21	0.09	12.89	0.89
27	4	6.20	0.51	2.47	0.07	15.71	0.73	19.69	3.71
28	3	6.71	0.13	2.65	0.02	17.54	0.38	23.38	0.07
29	3	7.13	0.08	2.83	0.08	19.37	0.56	28.23	1.15
30	3	7.66	0.14	3.04	0.04	23.15	0.55	27.40	0.16

**Table 3:** Sagittal and vertical diameters, projection surface area, and volume of the ossification centers of the left pubis

Gestational age (weeks)	Number of fetuses	Ossification centers of the left pubis							
		Sagittal diameter (mm)		Vertical diameter (mm)		Projection surface area (mm^2^)		Volume (mm^3^)	
		Mean	SD	Mean	SD	Mean	SD	Mean	sd
22	2	2.32	0.03	1.30	0.01	3.18	0.11	7.12	0.57
23	3	2.95	0.40	1.52	0.16	4.13	0.45	7.90	0.18
24	9	3.73	0.41	1.92	0.19	5.65	1.02	9.63	0.59
25	2	4.36	0.05	2.34	0.03	7.73	0.09	11.10	0.95
26	4	4.84	0.06	2.39	0.05	9.19	0.06	12.68	0.97
27	4	6.10	0.49	2.57	0.06	15.72	0.67	19.82	3.74
28	3	6.73	0.13	2.70	0.08	17.53	0.41	23.32	0.20
29	3	7.16	0.09	3.01	0.02	19.87	1.28	27.48	0.56
30	3	7.70	0.19	3.06	0.02	23.34	0.61	27.60	0.16

**Fig. 3:**
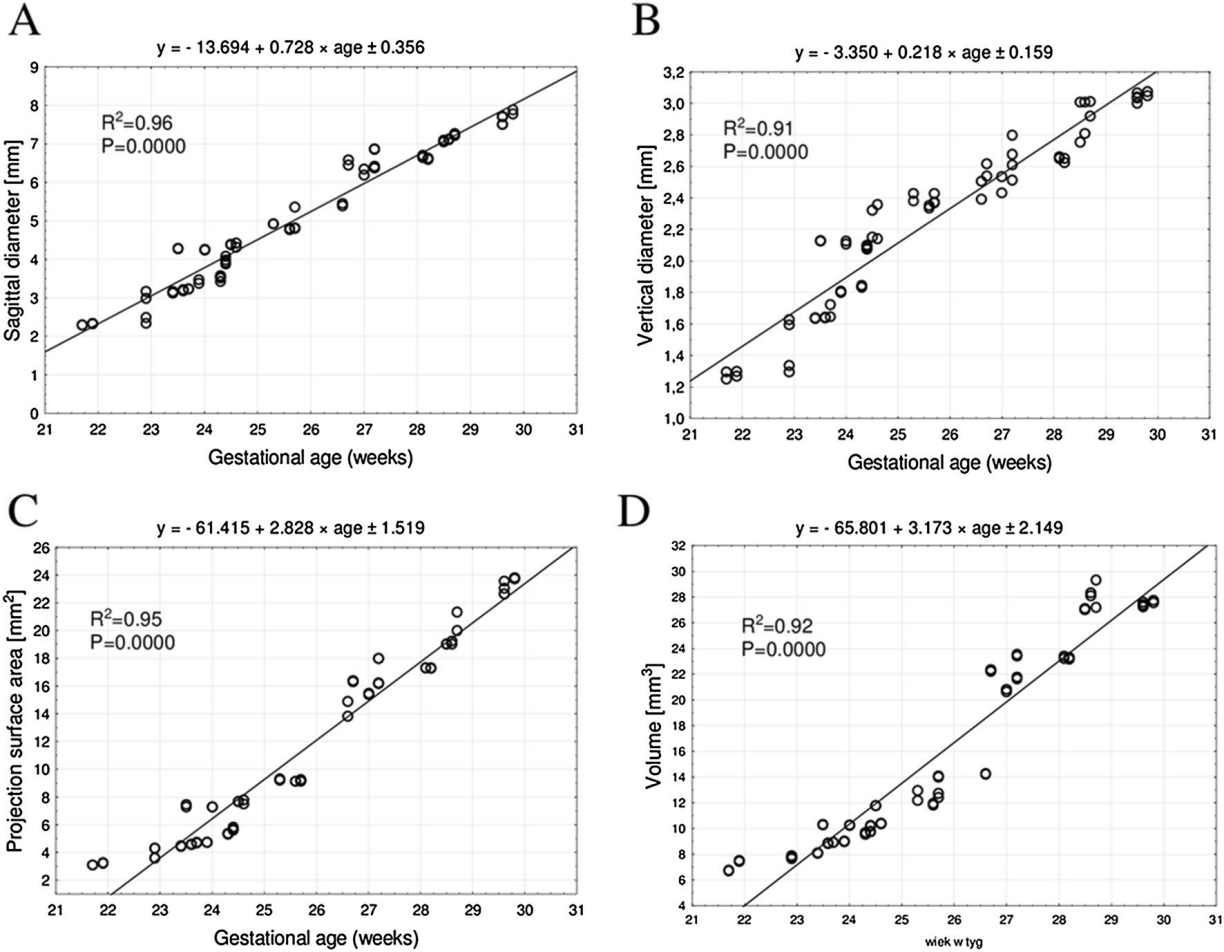
Regression lines for sagittal diameter (**a**), vertical diameter (**b**), projection surface area (**c**), and volume (**d**) of the pubic primary ossification center

Between weeks 22 and 30 of gestation, the mean sagittal diameter of the pubic ossification center was found to increase from 2.31 ± 0.03 to 7.66  ± 0.14 mm on the right side, and from 2.32 ± 0.03 mm to 7.70 ± 0.19 mm on the left side, following the linear function: *y* = − 13.694 + 0.728 × age ± 0.356 (*R*^2^ = 0.96), as presented in Fig. 3a.

The mean vertical diameter of the pubic ossification center ranged from 1.26  ± 0.01 mm at week 22 to 3.04 ± 0.04 mm at week 30 on the right side, and from 1.30 ± 0.01 to 3.04 ± 0.04 mm on the left side, according to the linear function: *y* = − 3.350 + 0.218 × age ± 0.159 (*R*^2^ = 0.91), as displayed in Fig. 3b.

The pubic ossification center revealed an increase in mean projection surface area from 3.17 ± 0.10 mm^2^ at week 22 to 23.15 ± 0.55 mm^2^ at week 30 on the right side, and from 3.18 ± 0.11 mm^2^ to 23.34 ± 0.61 mm^2^, respectively, on the left side, following the linear function: *y* = − 61.415 + 2.828 × age ± 1.519 (*R*^2^ = 0.95)—(Fig. 3c).

During the study period, the mean volume of the pubic ossification center increased from 7.09 ± 0.48 to 27.40 ± 0.16 mm^3^ on the right side, and from 7.12 ± 0.57 to 27.60 ± 0.16 mm^3^ on the left side, following the linear function: *y* = − 65.801 + 3.173 × age ± 2.149 (*R*^2^ = 0.92)—(Fig. 3d).

## Discussion

Among the three constituents of the hip bone, the pubis is the last to ossify, between months 5 and 6 of gestation. The primary ossification center is located within the superior pubic ramus, in front of the acetabulum and just in the close vicinity of the obturator and femoral nerves [4, 9, 10, 16, 24, 26]. During the early development, the pubic ossification center is dumbbell-shaped [24] or bean-shaped [4], and constitutes the smallest and most dainty component in the whole pelvis. At this stage, it has two ends: the lateral (hip) one, which is more rounded and pointed obliquely downwards, and the medial (flat) one, which is pointed straightly downwards; the latter is a presumptive pubic body [24].

Caffey and Madell [4] presented the entire pubis to ossify from only one ossification center which was subsequently fused with the iliac and ischial ossification centers. Contrariwise, Hess [15] identified four types of pubic ossification. In type A, the ossification center was confined to the superior pubic ramus, and the process of ossification did not progress medially toward the connection with the inferior pubic ramus. Thus, the medial end of the ossification center did not increase in size. In type B, the ossification center was dumbbell-shaped, with a narrow central part and two enlarged ends. The central part was located in the superior pubic ramus, with its ends pointed towards the pubic body and the connection with the ilium and ischium, and so, ossification did not progress towards the inferior pubic ramus. Type C was characterized by a hook-shaped ossification center, which caused ossification to occur towards the inferior pubic ramus. In type D, all the forenamed types of ossification centers can occur in one or both pubic bones. Since the distinction between types A–C is not explicit, one type can gradually transform into another. Caffey and Madell [4] demonstrated that at the time of birth, type B prevailed in both full-term and preterm neonates, constituting 52.9% and 61.9%, respectively, while type D was the rarest in both groups, amounting to 1.9% and 1.2%, respectively. Caffey and Madell [4] also noted the ossification process to be more advanced in female neonates.

At birth, the pubis is somewhat oval, with its articular surface pointed anteriorly [24] and with its superior pubic ramus considerably ossified [9]. Basing on 1286 radiographs of full-term neonates, Caffey and Madell [4] found that the superior pubic ramus was ossified in all examined cases. At the age of month 6, the ossification process of the superior pubic ramus advanced upwards, which resulted in the fusion with the ilium and the ischium. The fusion of the primary ossification centers of the pelvic bone first occurs between the ischium and the pubis [24, 26]. At birth, the pubis is still separated from the ischium by cartilage in the form of the ischiopubic synchondrosis [14, 20]. The age at which this synchondrosis starts to ossify ranges from 4 to 12 years [14, 20]. These two bones initially fuse only within their rami, while the fusion within the acetabulum starts as late as during puberty [24, 26].

It should be added that secondary ossification centers appear at the anterior edge of the pubic symphysis during puberty and fuse with the pubis during late adolescence or young adulthood [13, 26].

Francis [12] analyzed 640 radiographs of fetuses with CRL values ranging from 32 to 472 mm. By examining the primary ossification centers of the pelvis, the author showed that, in male and female fetuses with a CRL of less than 160 mm, no clear difference in the timing of these ossification centers could be observed. In turn, in fetuses with a CRL greater than 160 mm, the primary ossification centers in female fetuses could be noticed earlier than those in male fetuses. As regards the pubis, the primary ossification center was first noticed in a male fetus with a CRL of 156 mm and was located in the superior pubic ramus. In the group of fetuses with CRL values of 136–159 mm, it was the only fetus in which pubic ossification had commenced. In the subsequent five CRL range groups: 160–209 mm, 210–249 mm, 250–279 mm, 280–339 mm, and over 340 mm, the pubic ossification centers were observed in 43, 89, 83, 98, and 100% of the examined cases, respectively. According to the author, the primary ossification center of the pubis should be visible at a minimum CRL of 160 mm in female fetuses and at a CRL greater by a few milliliters in male fetuses. In our study, the pubic ossification center did not demonstrate sex or laterality differences, and was visible in fetuses of both sexes starting from week 22 of gestation. This finding was in line with studies conducted using CT for femoral [3] and iliac [2] ossification centers in human fetuses.

This paper is the first report to quantitatively evaluate the pubic ossification center in human fetuses using computed tomography and, concurrently, mathematical growth models. The pubic ossification center grew proportionately to fetal age in weeks in respect to its sagittal and vertical dimensions, and projection surface area and volume, as follows: *y* = − 13.694 + 0.728 × age ± 0.356, *y* = − 3.350 + 0.218 × age ± 0.159, *y* = − 61.415 + 2.828 × age ± 1.519 and *y* = − 65.801 + 3.173 × age ± 2.149, respectively. In a study of the development of the ilium’s ossification center, its growth dynamics regarding the vertical and sagittal dimensions followed the natural logarithmic functions: *y* = − 63.138 + 33.413 × ln(CRL) ± 1.609 and *y* = − 59.220 + 31.353 × ln(CRL) ± 1.736, respectively. In turn, the projection surface area and volume of the ossification center increased linearly: *y* = − 105.681 + 1.137 × CRL ± 16.035 and *y* = − 478.588 + 4.035 × CRL ± 14.332, respectively [2]. The obtained numerical data regarding the pubic primary ossification center can be useful in the diagnostics of skeletodysplasiae that are often characterized by a disrupted or delayed fetal growth.

In the literature, we have not found any reports concerning the dimensions of the pubic ossification center, which precludes a more comprehensive discussion on this topic.

Delay in the ossification process of the pelvis can be either generalized or isolated. Generalized delay is typical of endocrine disorders, cases of malnutrition, chronic diseases, and chromosomal aberrations. In turn, local delay involves a structural defect of one bone [26]. Diseases with abnormalities of the pubis include achondrogenesis types 1 and 2, campomelic dysplasia, cleidocranial dysplasia, spondylometaphyseal dysplasia, thanatophoric dysplasia, and hypophosphatasia [14, 26]. The delayed ossification process of the pubis and ischium cannot be detected by ultrasonography, but can be recognized due to MRI [21]. Moreover, high-quality prenatal radiography may complete and facilitate diagnosing skeletodysplasiae, but because of the difficulties in controlling the position of the in utero fetus is not used in routine examinations. Obviously, exposure to radiation is a potential threat to the normal development of the fetus. In dubious conditions, an MRI examination can provide additional information [21]. Achondrogenesis type 2 is manifested by the absence of ossification centers in vertebral bodies, and the pubis and ischium, as well as by the shortening of long bones. The prenatal ultrasonic diagnostics is an effective method of detecting those defects due to a high degree of shortening of the limbs and absence of ossification centers in the vertebral column [13]. Cleidocranial dysplasia is a disorder characterized by abnormalities in the vertebral column curvatures and cranial ossification, hypoplasia or dysplasia of the clavicle, delayed ossification of the pelvic bones, mostly the pubis, and abnormal ossification of shafts [8, 9]. Delayed ossification of the pubis causes an increase in the distance between the bilateral pubic bones, thus leading to numerous disturbances that can be misdiagnosed as urinary bladder exstrophy, epispadias, or other defects within the anterior abdominal wall [9].

Van Zalen-Sprock et al. [23] compared three imaging methods, X-ray and ultrasonography performed abdominally and transvaginally, in detecting ossification centers in the fetal skeleton. The earliest finding of an ossification center could be done with the use of X-ray examinations. At the same time or in the following week, ossification centers were visible due to transvaginal ultrasonography. Abdominal ultrasonography allowed visualizing ossification centers 1 or 2 weeks later. Victoria et al. [25] and Cassart et al. [5] demonstrated the use of 3D CT to offer a higher imaging precision compared to 2D ultrasound with respect to skeletodysplasiae. Computed tomography eliminates the overlap between anatomical structures, and allows distinguishing between tissues and visualizing the examined structure in every plane and at any time without sacrificing image detail after the examination [22].

Currently, routine ultrasonic examinations are complemented by MRI. This examination is critical in the second and third trimesters of pregnancy, when ultrasonic imaging offers results that are either ambiguous or limited by, e.g., small volume of the amniotic fluid (oligohydramnios) or breech presentation of the fetus [7].

The use of MRI in fetal examinations refers mainly to congenital defects of the central nervous and skeletal systems, as well as congenital defects of thoracic and abdominal viscera [1]. The newly developed Cine-MRI techniques provide an innovative insight into the movements of the entire fetus in the three-dimensional environment of the uterus during pregnancy [24].

The main limitation of this study was a relatively narrow fetal age group, ranging from week 22 to 30 of gestation, and a relatively small study group, including 33 human fetuses.

## Conclusions

1. The size of the pubic primary ossification center shows neither sex nor laterality differences.
2. The growth dynamics of the vertical and sagittal diameters, projection surface area, and volume of the pubic ossification centers follow proportionately to fetal age.
3. The obtained numerical findings of the pubic ossification center are considered age-specific reference data with clinical implications in the diagnostics of congenital defects.
